# A Dark Future of Endangered Mountain Species, *Parnassius bremeri*, Under Climate Change

**DOI:** 10.1002/ece3.71178

**Published:** 2025-04-01

**Authors:** Kyung Ah Koo, Seon Uk Park

**Affiliations:** ^1^ Korea Environment Institute Sejong‐si Republic of Korea; ^2^ National Institute of Ecology Gyeongsangbuk‐do Republic of Korea

**Keywords:** climate change, conservation policies and measures, dispersal capacity, endangered mountain species, hybrid species distribution models, land‐use change

## Abstract

Climate and land‐use changes are key factors in the habitat loss and population declines of climate change‐sensitive endangered species. We assessed the climate change effects on the distribution of *Parnassius bremeri*, a critically endangered wildlife species in the Republic of Korea, in association with food availability ( *Sedum kamtschaticum* and *Sedum aizoon* ), land‐use change, and dispersal limitation. We first predicted the current and future distributions of *P. bremeri*, *S. kamtschaticum* , and *S. aizoon* using the presence/absence data and current (2000) and future climate data (2050, 2100) with BioMod2, an ensemble platform for species distribution model projections. Then, the dispersal capacity of *P*. *bremeri* and land‐use change were coupled with SDMs using MigClim. We used future climate and land‐use changes predicted according to the SSP scenarios (SSP1‐2.6, SSP2‐4.5, and SSP3‐7.0) and the dispersal model estimated from previous studies. The current distributional areas of *P*. *bremeri* were predicted to be about 10,956 km^2^ without land‐cover coupling and 8.861 km^2^ with coupling, showing land‐cover decreased by about 19% of the suitable habitat. The future predictions under climate change only showed the distribution reduced by 56% and 50% in 2050 and 2100 under SSP1‐2.6, respectively, 55% and 48% under SSP2‐4.5, and 44% and 14% under SSP3‐7.0. Applying land‐use change and dispersal capacity further decreased the future distribution of *P*. *bremeri* but trivially (about 0.42% on average). The strict conservation policies and measures for *P*. *bremeri*'s habitats explain the trivial additional decrease, delaying its habitat loss. However, our results suggest that such efforts cannot halt the climate change‐driven habitat loss trend of *P*. *bremeri*. Strong climate mitigation efforts and promoting the species' adaptive capacity are the only ways to reverse the tragic decline of climate‐sensitive species.

## Graphical abstract

We assessed the climate change effects on the distribution of *Parnassius bremeri*, a critically endangered butterfly species in the Republic of Korea, in relation to food availability (*Sedum kamtschaticum* and *Sedum aizoon*), land‐use change, and dispersal limitations. Our findings indicate that climate change is a significant driver of habitat loss for *P*. *bremeri*. Urgent climate mitigation efforts and strategies to enhance the species' adaptive capacity are essential to reversing this alarming trend.

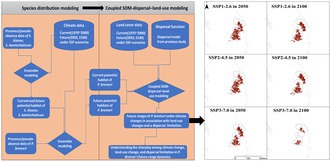

## 1 Introduction

Climate and land‐use changes are primary drivers of biodiversity loss (Newbold et al. 2016; Visconti et al. 2016). Climate change has shifted the geographical range, abundance, and seasonal activities of numerous species (Allen et al. 2018; Masson‐Delmotte et al. 2019), particularly influencing the life cycles and population dynamics of butterflies, with consequent horizontal and vertical changes in their ranges (Adhikari et al. 2020; Kwon et al. 2010, 2014; Lee et al. 2020). Land‐use changes, converting natural lands to urban and agricultural lands, have destroyed the habitats of numerous butterfly species (Islam and Weil 2000; Masayi et al. 2021; Uddin Mahtab and Karim 1992). Habitat loss is particularly severe for butterfly species that inhabit forest and grassland areas, accelerating their extinction rate (Novacek and Cleland 2001; Turvey and Crees 2019). Such effects of climate change and land‐use change on the range and populations of butterflies are species‐specific. While some studies have shown that habitat loss has a greater impact than climate change on the range and occurrence of butterflies with broad niche breadth (Kwon et al. 2021; Pollard and Yates 1993; Saarinen et al. 2003), others have reported that climate change is a major factor in changes in populations and ranges of butterflies particularly adapted to cold climate conditions (Halsch et al. 2021; Wanger 2020).

Changes in biotic interactions, including resource‐consumer interactions, and migration capacity in association with climate and land‐use changes, are known to affect the dynamics of species' range and population (Baer and Gray 2022; Dormann et al. 2018; Wisz et al. 2013). Incorporating data on biotic interactions into regional‐ to landscape‐scale species distribution models (SDMs) can significantly enhance model accuracy and provide insights into the factors driving species distributions (Baer and Gray 2022). For instance, the distribution and species richness of frugivorous birds in Africa have been shown to correlate with the diversity of food plants (Kissling et al. 2007). Likewise, for some butterfly species, the population carrying capacity can be predicted by the vegetation composition (Curtis et al. 2015). Dispersal capacity, the adaptation capacity of species, directly limits species' ability to shift to climatically suitable habitats (Koo and Park 2022). Dispersal capacity limits the range expansions of warm‐adapted species and increases the vulnerability of cold‐adapted species to climate warming (Koo and Park 2022; Morgan and Venn 2017).

*Parnassius bremeri* is distributed across countries in Far East Asia, such as the Republic of Korea (ROK), China, Japan, and Russia (National Institute of Biological Resources 2018a). *P*. *bremeri* inhabited the Korean peninsula in the past but is currently found in small restricted areas in eastern ROK (National Institute of Ecology 2022). Urban and agricultural developments destroy its main habitats, such as forest peripheral areas and grasslands, and shrink its range. Additionally, its population has declined due to overexploitation for ornamental purposes (National Institute of Biological Resources 2018a). Thus, *P*. *bremeri* has been protected by law since 1989, assessed as a vulnerable (VU) species in the Korean Red List, and classified as an endangered species in the Republic of Korea (Ministry of Environment, 2011). Previous studies on *P*. *bremeri* have focused on developing DNA markers and understanding the factors influencing its growth and geographical distribution (Kim et al. 2009; Lee et al. 2022; Park et al. 2017). Those studies included distribution pattern analyses, the characteristics of habitat utilization (Ko et al. 2004), metapopulation structure and migration (Kim et al. 2011), habitat connectivity (Kim et al. 2012), and the growth response to temperature stress (Park et al. 2017). However, the effects of climate and land‐use changes on the distribution of *P*. *bremeri* have only been studied at the local scale. For effective conservation, we need to understand the national‐scale responses of *P*. *bremeri* to those changes.

Species distribution models (SDMs) have been widely used in conservation biology, climate change studies, etc., due to easy construction processes, requesting relatively simple and small datasets (Erickson and Smith 2023; Franklin 2013; Guillera‐Arroita et al. 2015; Kaky et al. 2020; Sofaer et al. 2019). However, the SDMs based on the statistical correlations could not describe ecological processes and multiple factors' interactions among the target species and biotic and abiotic environments, and the model predictions, especially future predictions, include high uncertainty (Dormann et al. 2012; Thuiller et al. 2019). To overcome these drawbacks, hybrid models, combining SDMs with ecological processes such as dispersal, population dynamics, trophic interactions, etc., are applied to predict habitat suitability and distribution (Ellner and Fieberg 2003; Engler and Guisan 2009). Hybrid models improve the outcomes by combining SDMs with ecological mechanisms and processes, such as population dynamics (Acevedo et al. 2017; Franklin et al. 2014; Pagel and Schurr 2012), trophic interactions (Barber et al. 2021; Liu et al. 2023; Mellin et al. 2016), and dispersal processes (Koo and Park 2022; McClelland et al. 2020; Penteriani et al. 2019).

This study aimed to predict the effects of climate change on *P*. *bremeri* in interactions with land‐use changes, migration limitations, and food sources. For this, we predicted the potential habitats of *P*. *bremeri* in the current and future climate conditions and then the future change of its distributions under interactions among climate, food availability, land‐use changes, and dispersal capacity. The findings of this study would provide fundamental knowledge to set the direction of conservation policies and practices for *P*. *bremeri* and other endangered species.

## 2 Materials and Methods

**FIGURE 1:**
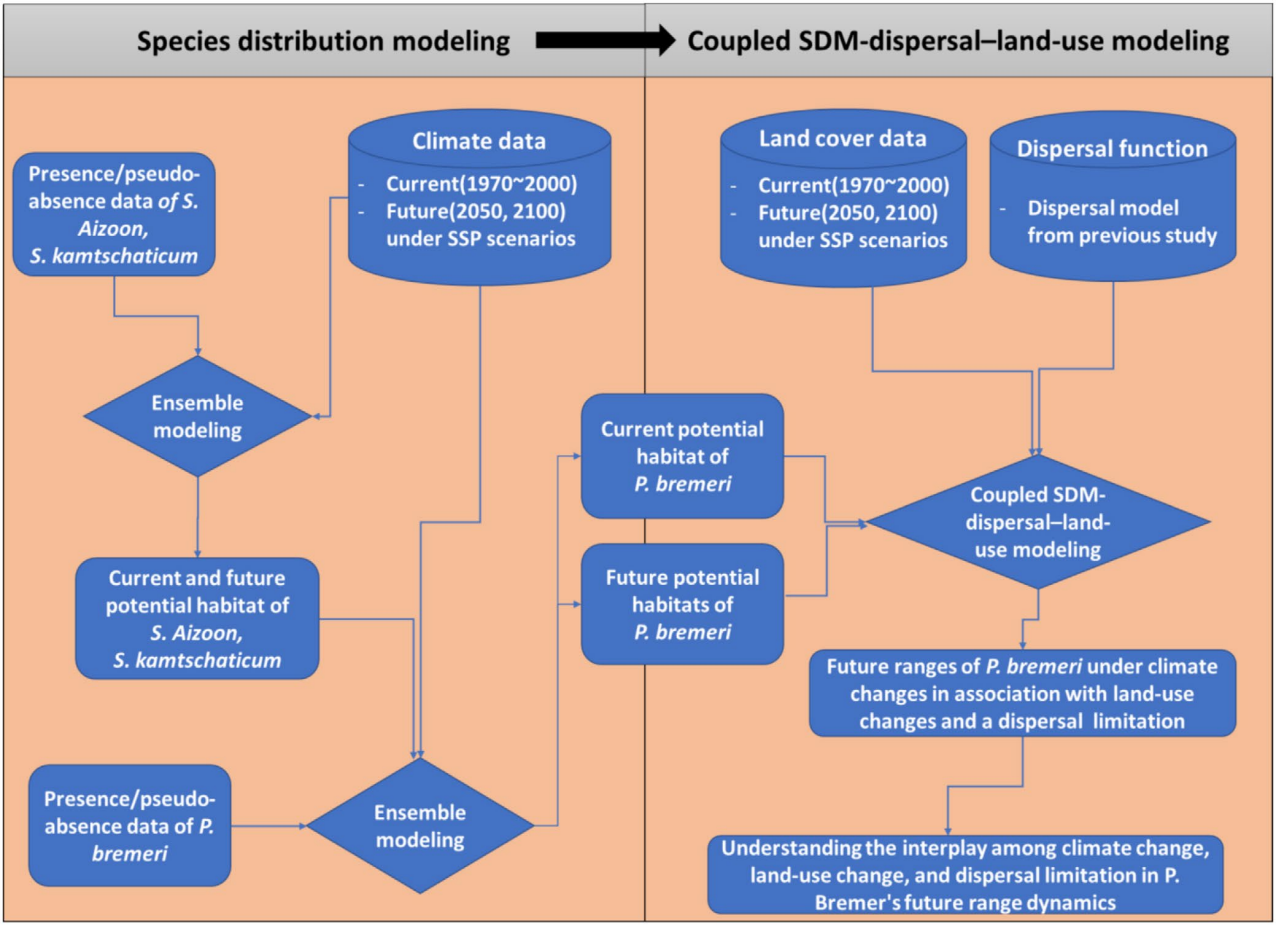
The study flowchart outlines the key steps from data preparation to modeling.

The following subsections describe the general information on the study species and sites and details of the key steps involved in the research process, from data preparations to modeling (Figure 1).

### 2.1 Study Species and Site

*P*. *bremeri* belongs to the Papilionidae family and is among the large varieties of the *Parnassius* genus butterflies (National Institute of Biological Resources 2018a). The following information on the biology and life cycles of *P*. *bremeri* is from Kim et al. (1999). *P*. *bremeri* goes through four life stages: egg, larva, pupa, and adult. From mid‐May to early June, it lays eggs on the branches of its food sources, such as the herbaceous plants of the *Sedum* genus, or in crevices of rocks. After hatching from the eggs between June and July, the larvae remain inside the egg during their first instar and overwinter. When the temperature exceeds 18°C, the larvae emerge and go through repeated overwintering in their larval form, growing until the fifth instar. To pupate, the larvae spin cocoons between late April and early May. After this period, adults emerge and are active from early May to mid‐June, with females beginning to lay eggs on the third day after emergence.

As reported through field studies and breeding experiments, the species mainly feeds on herbaceous plants of the *Sedum* genus (family Crassulaceae) ( *Sedum kamtschaticum* , *S. aizoon* , *S. middendorffianum*, *S. takesimense*, and *S. zokuriense*) (Kim et al. 1999). This species is distributed in the far eastern regions of Russia and the northeastern regions of China (National Institute of Biological Resources 2018a). In Korea, *P*. *bremeri* had been distributed throughout the Korean peninsula in the past; however, owing to the rapid decrease in natural habitats and population, *P*. *bremeri* is currently found in restricted areas, such as Samcheok‐si and Jeongseon‐gun in Gangwon‐do and Yeongju‐si and Uiseong‐gun in Gyeongsangbuk‐do (Figure 1) (National Institute of Ecology 2022) and is classified as a critically endangered wildlife species at national and global scales (Lee et al. 2022).

**FIGURE 2:**
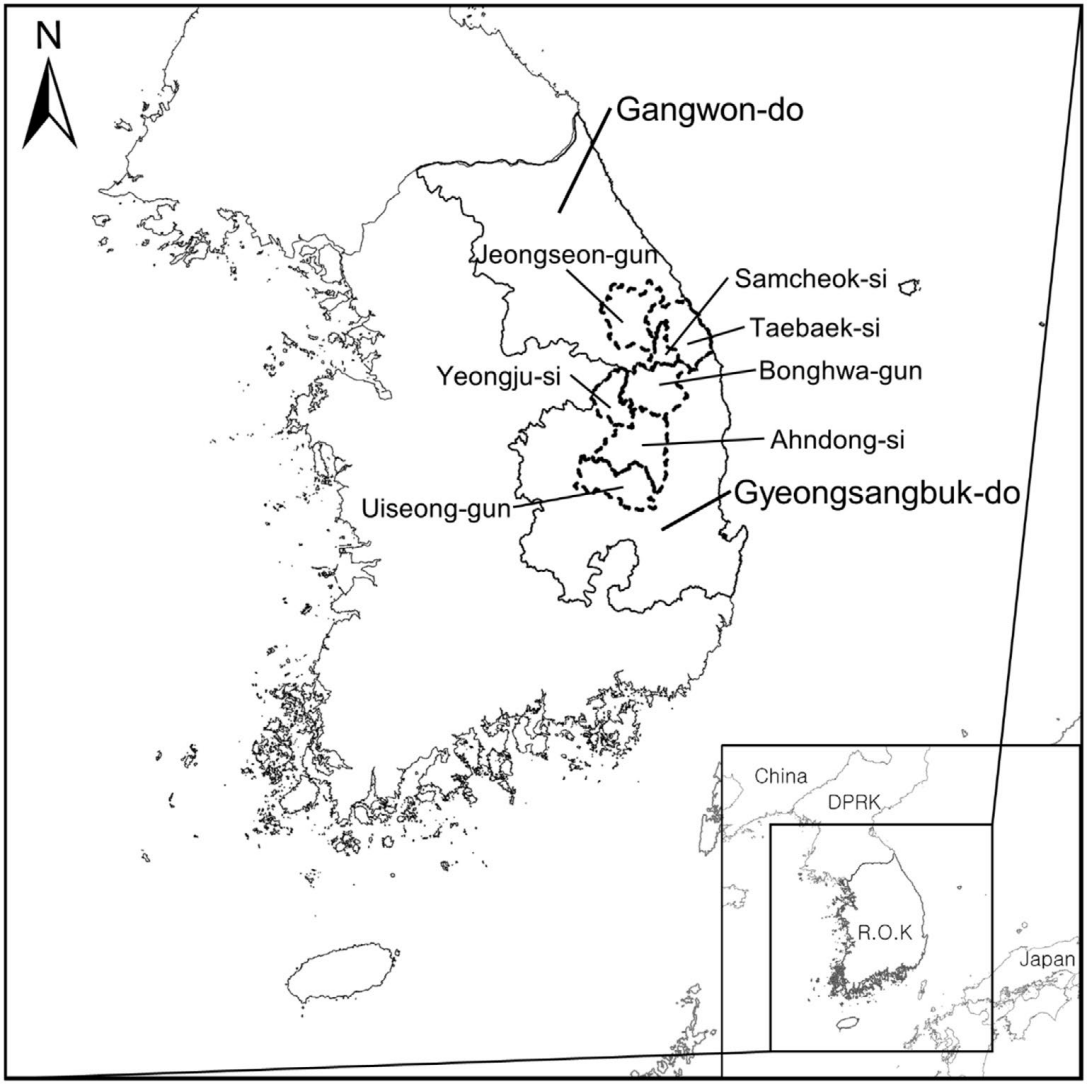
The Republic of Korea (ROK), the southern part of the Korean peninsula, is located in northeast Asia. The figure shows the important locations in the eastern ROK, which explains the habitats of *P. bremeri*.

The ROK is in the southern part of the Korean peninsula and in the eastern part of the Eurasian continent, surrounded by sea on three sides (Figure 2). The following information of ROK was summarized in The National Atlas of Korea 2nd Edition (National Geographic Information Institute 2020). The annual mean temperature of ROK, excluding island regions, is 7°C–15°C, and the annual precipitation reaches 1306.3 mm. According to the Köppen climate classification, ROK shows the temperate and polar climates. As of 2019, the proportion of national forests was 68.7%, agricultural lands 19.7%, and urban areas 5.6%. The urban area, in particular, showed a 2.6‐fold increase from 2.1% in 1989 to 5.6% in 2019. Most national forests are distributed in the Baekdudaegan mountain range in the eastern ROK due to rapid urbanization of the western ROK. The agricultural and urban areas are concentrated in the western ROK due to the East‐high and West‐low landscape of the ROK.

### 2.2 Species, Climate, and Land‐Cover Data for Modeling

The presence data of three species, *P*. *bremeri*, *S. kamtschaticum*, and *S. aizoon* , were collected from the National Survey on the distribution of endangered species (National Institute of Biological Resources 2012, 2015, 2018b, 2021) and the National Ecosystem Survey (second and fourth) (National Institute of Environmental Research 2006, 2013; National Institute of Ecology 2020). The duplicated data points within a grid of 30 arc sec (approximately 1 km^2^) were removed to eliminate the spatial autocorrelation. The presence data were 675 and 95 for the two prey species *S. kamtschaticum* and *S. aizoon* , respectively. For the SDM modeling, we generated the absences of 1000 and 400 datasets for *S. kamtschaticum* and *S. aizoon* , respectively, using a random sampling method. For the target species *P*. *bremeri*, the presence data were 74, and the absence data of 280 were generated with a random sampling method.

Numerous studies on the distribution of species have used 19 bioclimatic variables (Bioclim 01 to 19), which were developed as specialized climatic variables to describe the ecological processes of species and ecosystems (O'Donnel and Ignizio 2012). We selected six bioclimatic variables, including Bio01, Bio12, Bio03, Bio12, Bio13, and Bio14, because of the following reasons. First, a little ecophysiological research for *P*. *bremeri* was implemented in ROK, generally indicating sensitivity to a temperature condition (Park et al. 2017); thus, we selected these variables well accounting for the spatial characteristics of ROK's habitats in temperate regions. Second, we considered correlations among variables and accordingly selected variables, showing Pearson's correlation coefficients ≤ 0.7 (Table 1) (Koo et al. 2015). The current and future bioclimate data were obtained from the WorldClim website (http://www.worldclim.com/version2), and its spatial resolution was approximately 1 km^2^.

**TABLE 1:** The variables used in the development of SDM models for three species, *S. kamtschaticum, S. aizoon,* and *P. bremeri*.

Name	Definition	*S. kamtschaticum*	*S. aizoon*	*P. bremeri*
Bio01	Annual mean temperature	**○**	**○**	**○**
Bio02	Mean diurnal range [Monthly mean (max temp‐min temp)]	**○**	**○**	**○**
Bio03	Isothermality (BIO02/BIO7) (×100)	**○**	**○**	**○**
Bio12	Annual precipitation	**○**	**○**	**○**
Bio13	Precipitation of wettest month	**○**	**○**	**○**
Bio14	Precipitation of driest month	**○**	**○**	**○**
BFS_01	Predicted distribution ranges of *S. kamtschaticum*			**○**
BFS_02	Predicted distribution ranges of *S. aizoon*			**○**

*Note:* The predicted distribution ranges of two food source species, *S. kamtschaticum* and *S. aizoon* , were applied to develop the SDMs for the target species, *P. bremeri*.

We used the average of 1970 to 2000 for the current bioclimatic data (Fick and Hijmans 2017). We applied future bioclimatic data predicted by ACCESS‐CM2 (Bi et al. 2020), INM‐CM4‐8 (Volodin 2021), and UKESM1‐0‐LL, which are the General Circulation Model (GCM) corresponding to the CMIP6 (Meuriot et al. 2021). ACCESS‐CM2 (ACC) was developed by the Commonwealth Scientific and Industrial Research Organization in Australia, INM‐CM4‐8 (INM) by the Institute for Numerical Mathematics in Russia, and UKESM1‐0‐LL(UKE) by the Met Office Hadley Centre in the United Kingdom. INM was chosen because it was developed in a neighboring country, which reduces the increase in model prediction uncertainty with increasing distance due to its proximity to the initial simulation location (Jose et al. 2022). ACC and UKE were chosen because the Ministry of Environment, ROK, tested and used these models to predict future climate. The future climates in the 2050s (the average of 2041–2060) and 2100 s (the average of 2081–2100) were obtained by averaging the predictions of those three GCMs. Using the average generally reduced the model prediction uncertainty caused by individual models. The future climates were predicted based on the SSP scenarios newly presented in the sixth IPCC report: SSP1‐2.6, SSP2‐4.5, and SSP3‐7.0 (Allan et al. 2021; Allen et al. 2018). The three scenarios have the following assumptions: Environment‐friendly and sustainable economic growth with technological advances in renewable energy and minimal fossil fuel use in SSP1‐2.6; mitigated climate change and mid‐stage socioeconomic advancement in SSP2‐4.5; a social structure vulnerable to climate change due to delayed technological advancement and passive climate change policies in SSP3‐7.0.

The land‐use changes were considered for a more realistic prediction of *P*. *bremeri* distribution under future climate conditions. For this, the current (published in 2010, covering 2000 to 2009) and future (2050 and 2100) land‐cover maps were obtained from the National Environmental Information Network System (NEINS; https://www.neins.go.kr/Index) and the previous study (Song et al. 2018), respectively (Table S1 and Figures S1 and S2 in Appendix S1). Similar to the scenarios for climate change predictions, the future land‐cover maps were predicted under the assumptions of SSP1, SSP2, and SSP3 scenarios (Song et al. 2018). The SSP scenarios reflect population changes, climate change adaptation, greenhouse gas reduction, and socioeconomic advancements, such as changes in land use and energy consumption in the future.

### 2.3 Species Distribution Modeling

Suitable habitats with SDMs are estimated based on the statistical relationship between the presence–absence of target species and the environment, and the ensemble model has been applied to increase the model's predictive power(reference). Species distribution modeling was implemented using the BioMod2 R package, an ensemble platform for SDM projections (Thuiller et al. 2016). SDMs were produced by ensembling the nine individual model projections, including the Generalized Linear Model (GLM), Generalized Additive Model (GAM), Maximum Entropy Model (MaxEnt), Random Forest (RF), Generalized Boosting Model (GBM), Classification Tree Analysis (CTA), Artificial Neural Network, Surface Range Envelop (SRE), Flexible Discriminant Analysis, and Multiple Adaptive Regression Splines. Species distribution modeling was implemented using the 186 BioMod2 R package, an ensemble platform for SDM projections (Thuiller et al. 2016).

The presence–absence data were randomly split in a 7:3 ratio to develop and validate the SDMs. To minimize the bias related to the data split process, the process was repeated 20 times to construct 20 training datasets and 20 testing datasets. A total of 180 SDMs were developed based on the nine SDM algorithms and 20 training datasets (Guisan and Zimmermann 2000). The Area Under the Curve (AUC) of the Receiver Operating Characteristic and True Skill Statistic (TSS) were calculated with the test dataset to estimate the model predictive accuracy (Allouche et al. 2006; Jiménez‐Valverde 2012). The TSS value‐weighted ensemble models of *P. bremeri*, *S. kamtschaticum* , and *S. aizoon* were developed with the following equation (Equation 1) using individual models with the TSS value ≥ 0.7 for *P. bremeri*, ≥ 0.3 for *S. kamtschaticum* , and ≥ 0.5 for *S. aizoon* (Thuiller et al. 2019). The habitat suitability and distribution of the three species were predicted in the current and future climate conditions of the 2050s and 2100 s. Their distributions were determined by the probability of presence that presented the maximum predictive power of the ensemble SDMs, the maximum TSS value (Koo and Park 2022). The current and future distributions of *S. kamtschaticum* and *S. aizoon* were applied to the SDM modeling of *P. bremeri* as the biotic conditions, such as food availability, in current and future climate conditions:

(1) $$P_{\mathrm{TWE}}=\sum \frac{{\mathrm{TSS}}_{i}P_{i}}{{\mathrm{TSS}}_{i}}$$

where *i* is an individual SDM, *P*_*i*_ is the calculated suitability of distribution in the *i* SDM, TSS_*i*_ is the TSS for the SDM *I*, and *P*_TWE_ is the ensemble prediction of the TSS weighting for the target species.

### 2.4 Coupled SDM‐Dispersal–Land‐Use Modeling

The prediction of future distribution with the traditional SDMs is partially realistic due to the lack of consideration of other factors and processes, including land‐use change and migration capacity (Guisan and Thuiller 2005). Hence, the hybrid models incorporating additional biological processes (dispersal, population fluctuations, biological interactions, etc.) and other factors (land‐use change, pollution, etc.) have been used to enhance the reality of future predictions (Brotons et al. 2012; Rodríguez et al. 2019, Singer et al. 2018; Zurell 2017). We used MigClim, a hybrid model coupling a dispersal model and land‐use changes with SDMs, to predict more realistic distributions of *P. bremeri* under climate change (Engler and Guisan 2009; Engler et al. 2012). For the MigClim modeling, we need two types of baseline maps: the current and the future distribution maps obtained from SDM modeling, the current and future land‐cover maps, and a dispersal model. The land‐cover maps were reclassified into two categories, suitable and unsuitable, based on the information on their suitable habitat conditions (Kim et al. 1999).

The baseline maps were prepared for *P. bremeri* by combining the SDM projections with the reclassified maps. For a dispersal model, the migration distance was set according to a previous study that conducted a field experiment on *P. bremeri*; the maximum dispersal distance was 1.4 km per year (Kim et al. 2012). MigClim modeling also considered the barriers to migration; thus, we set the barriers where land covers were unsuitable habitats for *P*. *bremeri*. Forests and grasslands were set as suitable habitats, while other land covers, including agricultural lands, wetlands, barren land, urban areas, and water bodies, were barriers to *P. bremeri* migration. We ran the model 20 times and averaged them to minimize the prediction uncertainty.

## 3 Results

### 3.1 Model Accuracy and Variable Importance

**FIGURE 3:**
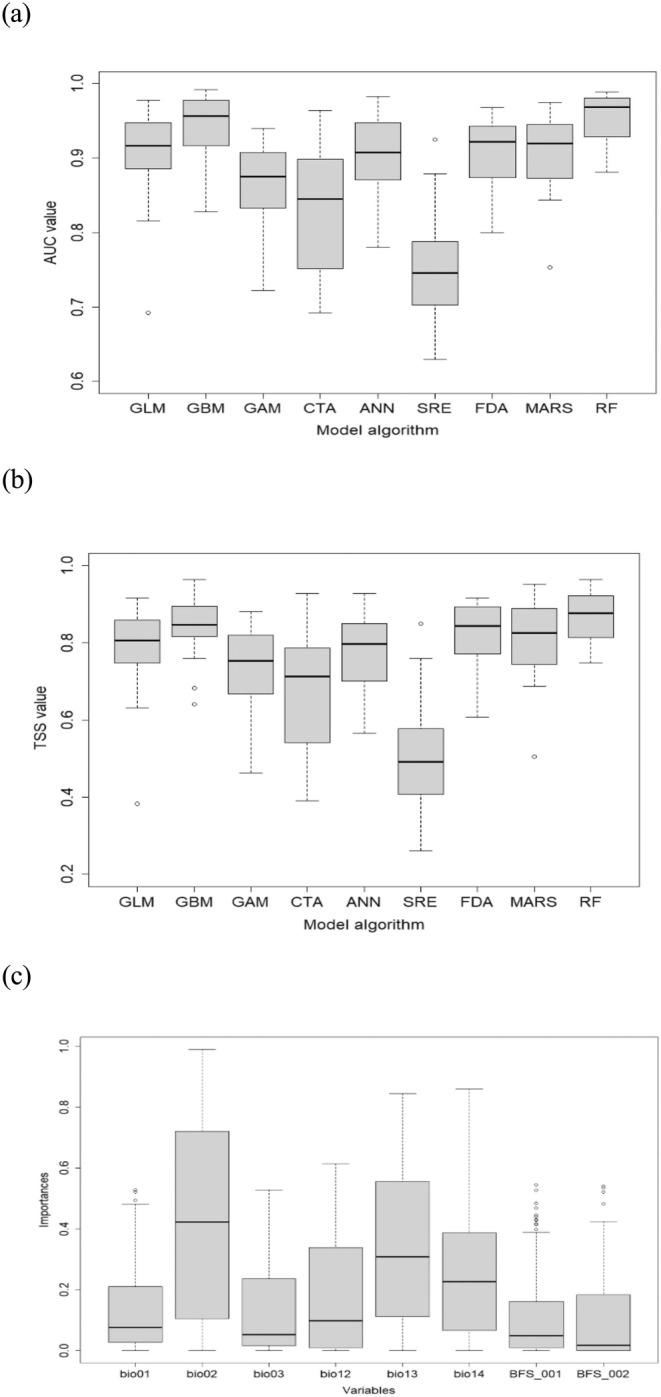
The AUC (a) and TSS (b) of the individual SDMs developed for *P. bremeri*. (c) Shows the importance of environmental variables for the *P. bremeri* models.

**FIGURE 4:**
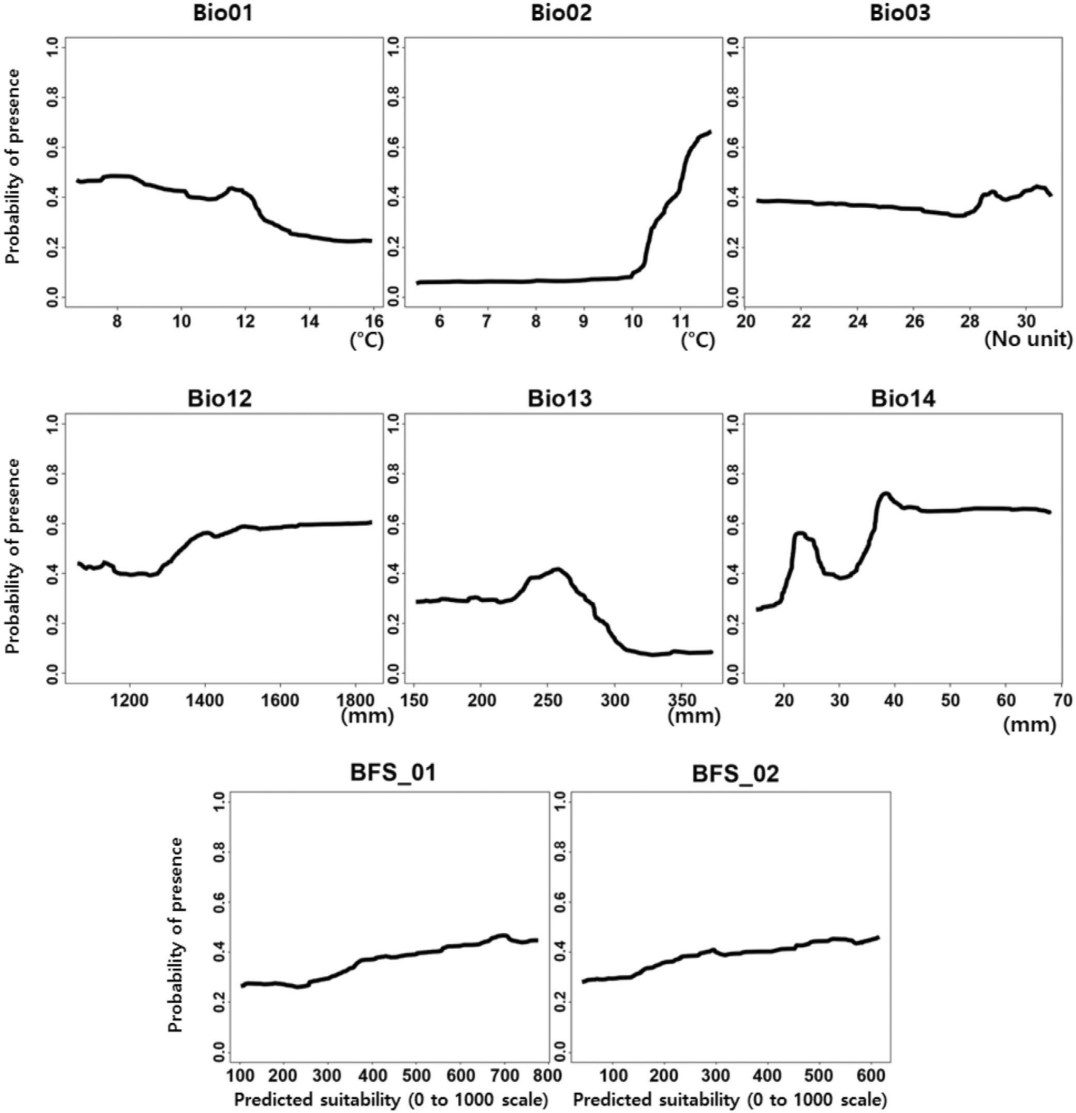
The response curves of the influencing variables on modeling the distribution of *P. bremeri*. Abbreviations were explained in Table 1.

The TSS value‐weighted ensemble model was developed for *S. kamtschaticum* with 147 individual SDMs (TSS ≥ 0.3) out of the 180 (Figure S3 in Appendix S1), *S. aizoon* with 31 individual SDMs (TSS ≥ 0.5) (Figure S5 in Appendix S1), and *P. bremeri* with 128 individual SDMs (TSS ≥ 0.7). The TSS value of 0.3 for *S. kamtschaticum* was lower than the values of the other two species, but this value was the maximum value that can produce the distribution map. It was because the TSS values of most individual SDMs for *S. kamtschaticum* were lower than 4 and around 3. The accuracies of ensemble SDMs for *S. kamtschaticum* and *S. aizoon* were as follows: TSS = 0.529 and 0.634, respectively; AUC = 0.845 and 0.896, respectively. The ensemble model of *P. bremeri* showed strong predictive power, having a high TSS value of 0.997 and an AUC of 0.975. The accuracy assessment of individual SDMs for *P. bremeri* showed that the predictive accuracies of GBM and RF were relatively higher than those of SRE, CTA, and GAM (Figure 3a,b). The variable importance assessment was implemented for *P. bremeri*'s 128 individual SDMs, composing the ensemble model. The result showed that the mean diurnal range (Bio02), precipitation of the wettest month (Bio13), and precipitation of the driest month (Bio14) were more influential than annual mean temperature (Bio01), Isothermality (Bio03), total precipitation (Bio12), and the distributions of two host plants ( *S. kamtschaticum* and *S. aizoon* ) in ensemble projection of *P. bremeri* distribution (Figure 3c). The response curves of influential variables, Bio02, Bio13, and Bio14, to the probability of *P. bremeri*'s presence were shown in Figure 4.

### 3.2 Predictions on the Distribution of *P. bremeri* Under Current and Future Climate Conditions

**FIGURE 5:**
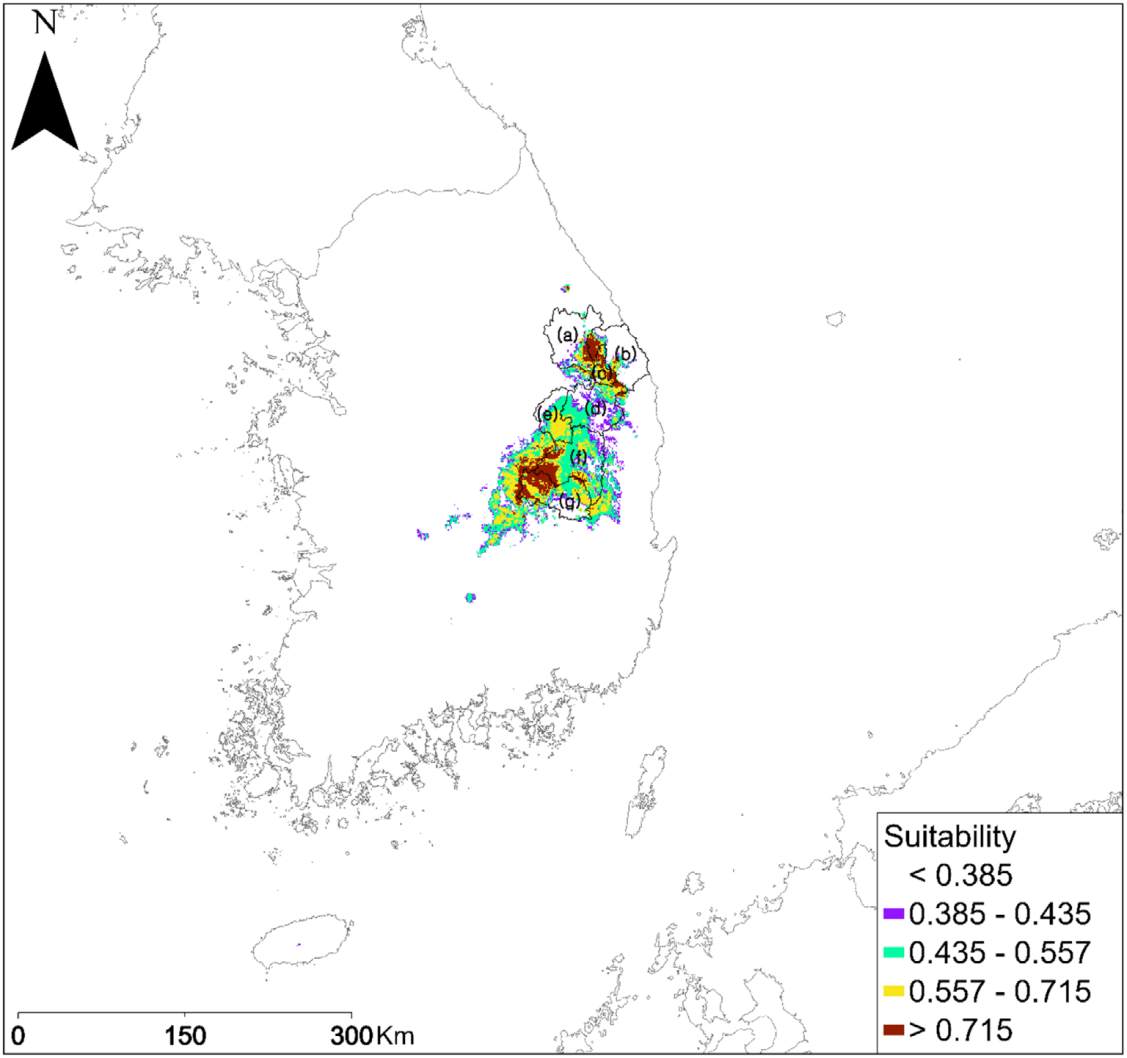
Predicted potential habitat of *P. bremeri* in current climate conditions: (a) Location name of Jeongseon‐gun, (b) Samcheok‐si, (c) Taebaek‐si, (d) Bonghwa‐gun, (e) Yeongju‐si, (f) Angdong‐si, and (g) Uiseong‐gun.

**FIGURE 6:**
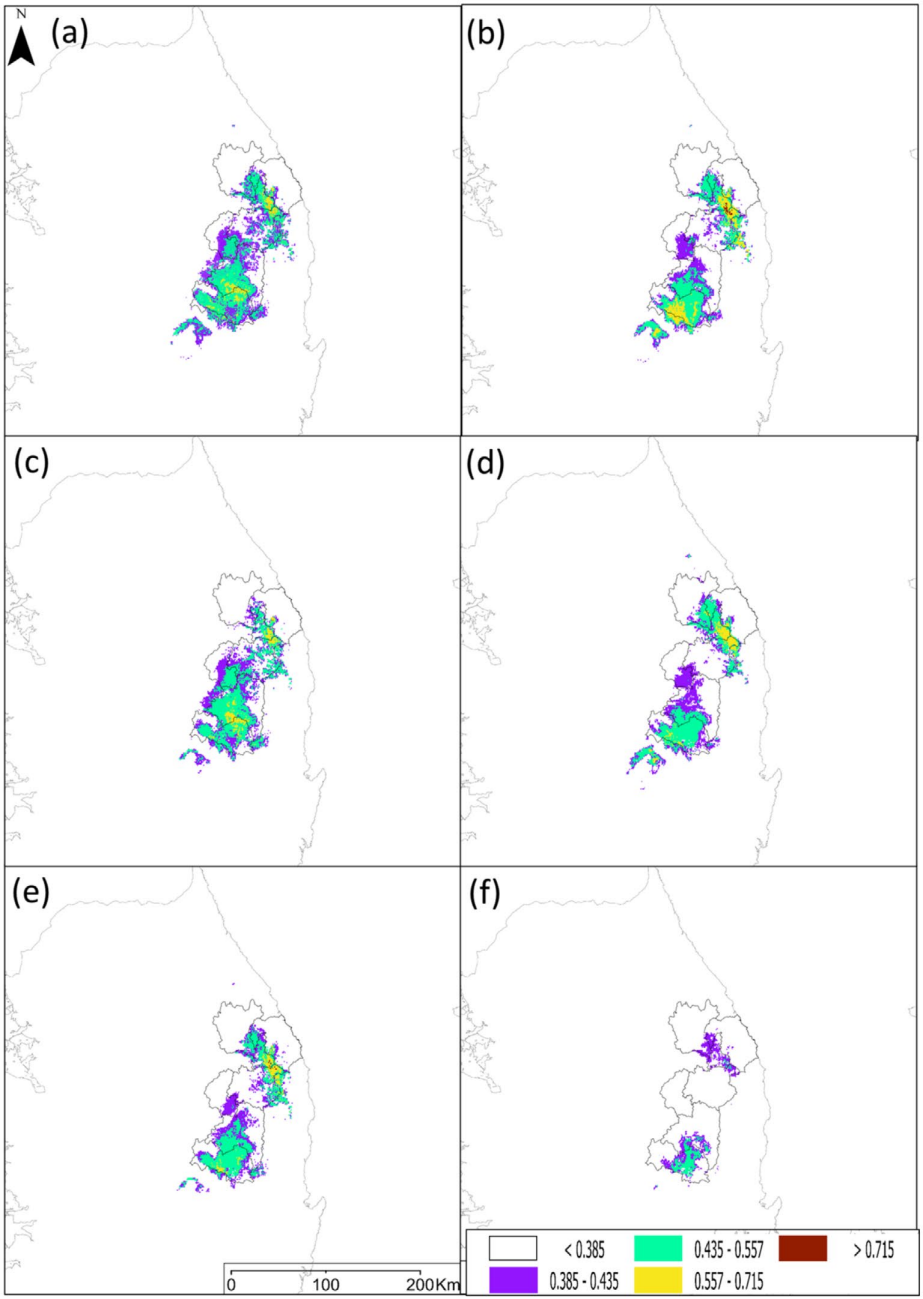
Predicted change in the future potential habitat of *P. bremeri* according to the climate change scenarios (SSP): (a) Presents the prediction under SSP1‐2.6 in 2050, (b) SSP1‐2.6 in 2100, (c) SSP2‐4.5 in 2050, (d) SSP2‐4.5 in 2100, (e) SSP3‐7.0 in 2050, and (f) SSP3‐7.0 in 2100.

The current and future climatic habitat suitability of *S. kamtschaticum* and *S. aizoon* were projected with the ensemble models under three climate change scenarios (Table S2 and Figures S3–S6 in Appendix S1), and then applied for *P. bremeri*'s current and future predictions. The current climatically suitable habitat of *P. bremeri* was estimated as 10,956 km^2^ (Figure 5). The suitable habitats of *P. bremeri* were mostly distributed in southeastern Korea and some areas of central Korea. The future habitats in 2050 were predicted as 6145, 6017, and 4799 km^2^ under SSP1‐2.6, 2–4.5, and 3–7.0, respectively, and the suitable habitats in 2100 as 5481, 5278, and 1572 km^2^ under SSP1‐2.6, 2–4.5, and 3–7.0, respectively (Figure 6). The 2050 predictions showed that the suitable habitats shrunk by about 44%, 45%, and 56% under SSP1‐2.6, SSP2‐4.5, and SSP3‐7.0, respectively, and the 2100 predictions by 50%, 52%, and 86% under SSP1‐2.6, SSP2‐4.5, and SSP3‐7.0, respectively.

### 3.3 Predictions of Future Distributions Under Interplay Among Climate Change, Land‐Use Change, and Dispersal Limitation

**FIGURE 7:**
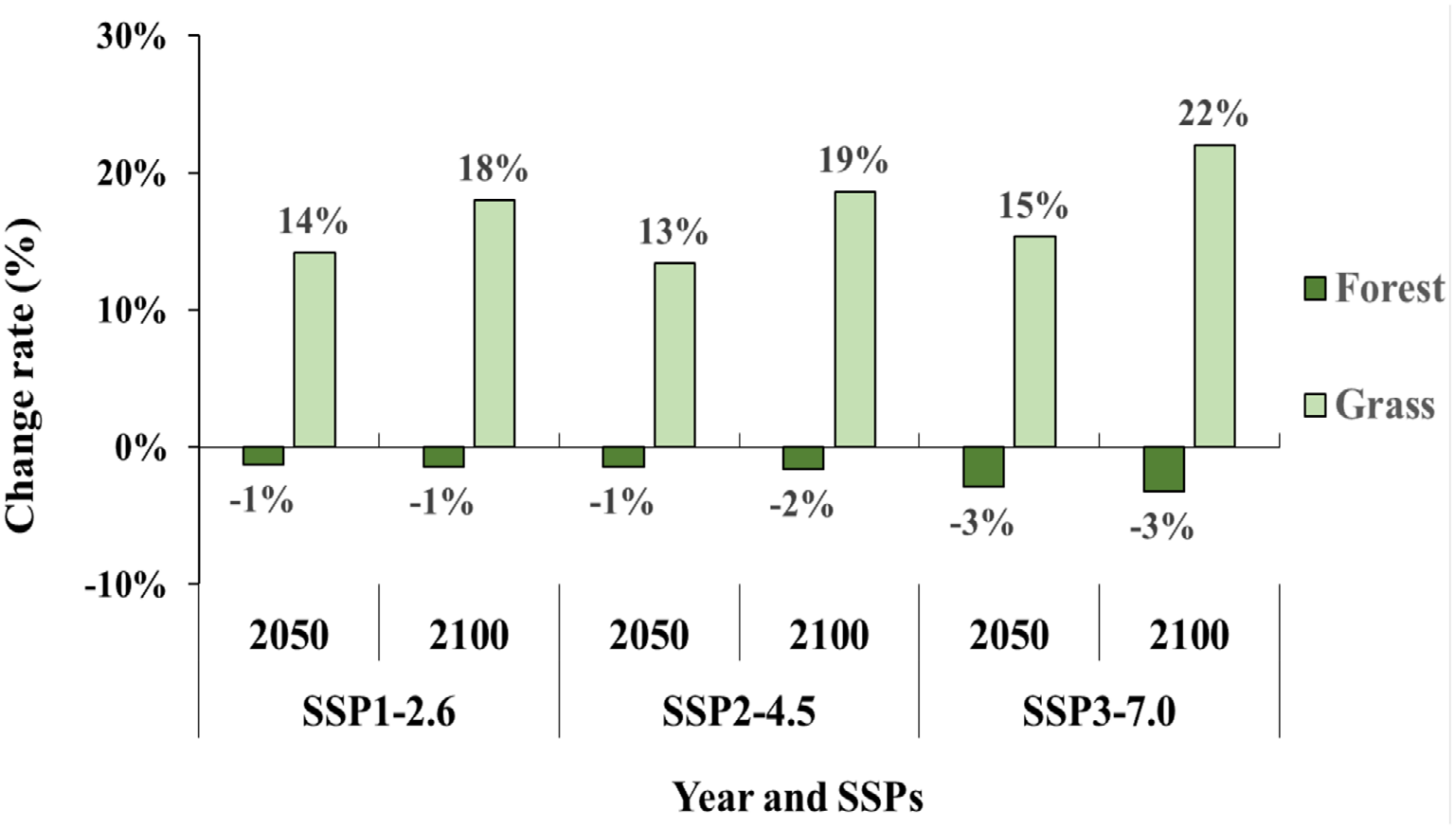
The rate of areal changes of forest and grassland in the future, 2050 and 2100, against their current, 2000, areas under SSPs. The forests and grasslands were classified as habitable habitats for *P. bremeri*.

**FIGURE 8:**
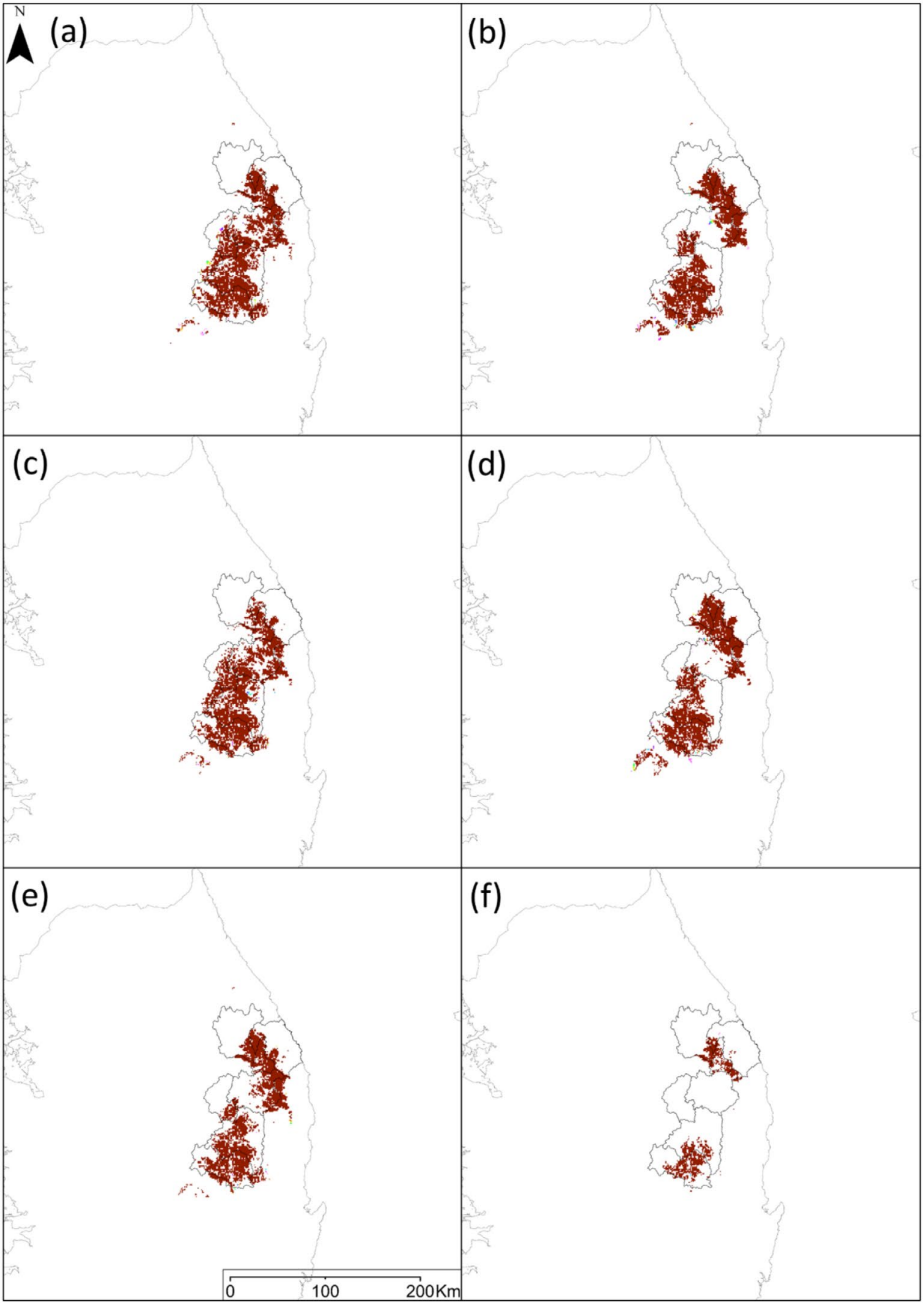
Predicted change in the future distributions of *P. bremeri* according to the climate change scenarios (SSPs) in association with land‐use change scenarios (SSPs) and dispersal capacity: (a) Shows the prediction under SSP1‐2.6 in 2050, (b) SSP1‐2.6 in 2100, (c) SSP2‐4.5 in 2050, (d) SSP2‐4.5 in 2100, (e) SSP3‐7.0 in 2050, and (f) SSP3‐7.0 in 2100.

A hybrid model developed for *P. bremeri* predicted its future distributions under climate changes in interaction with its dispersal capacity (1.4 km) and land‐use changes projected based on SSP1, SSP2, and SSP3 (Figure 8). Compared with the current land cover in 2010, grassland, a major land cover for *P. bremeri*, increased to an average of 16%, with a range of 13% to 22%, whereas forest decreased to an average of 2%, with a range of 1% to 3% under future climates (Figure 7). The most changes in forest and grassland areas were commonly predicted in 2100 under SSP3‐7.0.

The current distributional areas of *P. bremeri* coupled with the land cover map were predicted to be about 8861 km^2^, showing landscape configuration decreased about 19% of *P. bremeri*'s climatically suitable habitats. The current range coupled with the land‐cover map was used as a baseline for predicting the change rate of future distributions in association with land‐use change and dispersal limitations. Overall, climate changes shrank the range of *P. bremeri*, depending on the SSP scenarios, and land‐use change and dispersal limitation increased the habitat loss of *P. bremeri*. The average of the future ranges was predicted to decrease around 53%, ranging from 44% (all 2050 predictions under SSP1‐2.6 climate change scenarios in combination with land‐use change under SSP1 and/or dispersal capacity) to 86% (all 2100 predictions under SSP3‐7.0 climate change scenarios in combination with land‐use change under SSP3 and/or dispersal capacity), under all three cases. The average decrease of the future ranges was 52.82% under climate change only, 52.93% under climate change coupled with land‐use change, and 53.24% under climate and land‐use changes with dispersal limitation. The difference in decrease rate among the three cases was trivial; land‐use change increased the decrease rate by about 0.11% and land‐use change and dispersal limitation together by 0.42%. In detail, the decreases by land‐use change were only predicted as 0.29% in 2050 under SSP2‐4.5 and 0.12% in 2100 under SSP3‐7.0. The further decreases by dispersal limitation were shown as 0.47% and 0.69% in the 2050 and 2100 predictions under SSP1‐2.6, respectively, 0.29% in 2050 under SSP2‐4.5, and 0.12% in 2100 under SSP3‐7.0.

## 4 Discussion

We assessed the impact of climate and land‐use changes on the distribution of *Parnassius bremeri*, a critically endangered species in South Korea, in relation to food availability ( *Sedum Kamtschaticum* and *Sedum aizoon* ), land‐use change, and dispersal limitations. Our research found that (1) the mean diurnal range (Bio02), precipitation of the wettest month (Bio13), and precipitation of the driest month (Bio14) were influential factors to determine habitat suitability and distribution range of *P. bremeri* and (2) climate change was the single dominant factor of future habitat loss, showing reductions of 56% (2050) and 50% (2100) under SSP1‐2.6, 55% and 48% under SSP2‐4.5, and 44% and 14% under SSP3‐7.0. Land‐use change and dispersal capacity had minimal influence, contributing to a slight further reduction in the species' future distribution, averaging about 0.42%.

Our ensemble model predictions in 2050 and 2100 under climate‐only scenarios revealed that the climatically suitable habitats of *P. bremeri* would decrease mostly by losing the edges of their current habitats (Figure 5). The loss of their distributional edges led to habitat fragmentation under all SSP scenarios in 2100 and the SSP3‐7.0 scenario in 2050 (Figure 5b,d–f). The overall habitat suitability for *P. bremeri* was also predicted to be degraded under the assumptions of all SSP scenarios. In particular, high suitability areas (> 0.715) disappeared in 2050 and 2100 under all SSP scenarios due to the decreases of high suitability to either mid (0.435–0.557) or low (0.385–0.435) levels (Figure 5).

The hybrid model predictions under interplays among climate change, land‐use change, and dispersal capacity indicated that *P. bremeri* lost their habitats mostly under climate change, increasing the amount of habitat loss according to the climate change scenarios (Figures 2 and 8). *P. bremeri* lost most of their habitats by 2100 under the SSP3‐7.0 scenario, only showing the remaining habitats in Samcheck‐si and Taebaek‐si of Gangwon‐do and Ahndong‐si and Uiseong‐gun of Gyeongsangbuk‐do. The habitats of *P. bremeri* located in northeastern ROK (Samcheck‐si and Taebaek‐si in Gangwon‐do) and southeastern ROK (Ahndong‐si and Uiseong‐gun in Gyeongsangbuk‐do) kept connecting by the 2050s under the SSP1‐2.6 and SSP2‐4.5 scenarios but not in the 2100 s (Figure 8a–d). The predictions presented the habitats of two regions started disconnecting in 2050 under SSP3‐7, and the disconnectivity increased by 2100 under all three scenarios, maximizing under the SSP3‐7.0 scenario (Figure 8d,e).

We analyzed the interactive effects of species adaptation capacity, human disturbances, and climate changes on the future distributions of *P. bremeri*. The adaptation capacity was examined by applying dispersal capacity and human disturbance by land‐use changes, which described the level of habitat destruction and fragmentation. The results showed that, compared to the predicted range under the climate change‐only scenario, the dispersal capacity of *P. bremeri* decreased by 0.12% to 0.69%, depending on the SSP scenarios, for the future range. Unlike the dispersal capacity, land‐use changes only cause an average of 0.09% additional decrease in the future range of *P*. *bremeri*. Considering land‐use change has been a critical or dominant factor in determining species' future distributions, particularly near‐future predictions (Hansen et al. 2001; Jetz et al. 2007; Koo and Park 2022; Newbold et al. 2020; Sales et al. 2020; Williams and Newbold 2020), our predictions provide us with an important insight. Most habitats of *P. bremeri* are protected areas preserved by strong laws and policies. Our result supports, thus, that the protected area is an important and valuable measure for endangered species conservation (Brooks et al. 2004; Chowdhury et al. 2023; Gillingham et al. 2015; Kearney et al. 2020; Le Saout et al. 2013), delaying their habitat loss in a changing climate.

Butterflies have a short lifespan and are sensitive to environmental changes, making them widely used to estimate the impact of climate change on ecosystems (Dennis et al. 2003; Nadeau et al. 2017). Approximately 29% of studies employed butterflies in assessing the effects of climate on insects (Andrew et al. 2013). Previous studies showed that climate change, including temperature increases, more variable weather patterns, more extreme weather events, and increased water stress, was a major factor in declining butterfly populations, especially the populations of montane and univoltine butterfly species (Halsch et al. 2021; Wanger 2020). Such butterfly species, including *P. bremeri*, act as pollinators for many flowering plants, contributing to those plants' reproductive success and supporting the stability and resilience of montane ecosystems by helping maintain plant diversity and the related food webs (Ghazanfar et al. 2016). The loss of those butterfly species can disrupt food webs, having cascading effects on the stability and resilience of whole montane ecosystems. In addition, each butterfly species represents unique genetic diversity, indicating potential adaptations to changing environmental conditions (Mackintosh et al. 2019). Losing montane butterfly species, including *P. bremeri*, reduces the overall genetic diversity and the resilience to disturbances of montane ecosystems under a changing climate. Therefore, understanding and predicting the extent of the impact of climate change and establishing species‐specific measures accordingly for endangered montane butterfly species, such as *P. bremeri*, is crucial not only for the survival of those species but also for maintaining biodiversity and the health and resilience of ecosystems (Hill et al. 2021).

Our findings, which show that climate change—particularly shifts in the mean diurnal temperature range and changes in precipitation patterns of the wettest and driest months—will be a dominant factor in determining the future distribution of *P. bremeri* (a high‐altitude mountain species), strongly support the conclusions of previous studies. The future distributions of *P. bremeri* were predicted to decrease dramatically by approximately 52.82% under climate change. Our predictions also suggest that the current and future conservation practices and policies, including protected areas, increasing habitat connectivity, and restoring destructed and fragmented habitats, are unfortunately insufficient for preserving endangered species, particularly those most threatened by a changing climate. Therefore, we need more transformative and active management plans and measures directly related to strong climate change mitigation and adaptation strategies. These plans and measures include identifying climatic refuges in high‐latitude and high‐altitude mountain areas for *P. bremeri* and assisting their migration to these refuges. Additionally, they involve establishing ex‐situ conservation institutions, along with implementing robust climate change mitigation strategies, such as raising the Nationally Determined Contribution (NDC) targets and promoting activities like increasing the share of renewable energy sources.

Identifying the extent of climate change impacts on endangered butterfly species relies on the model's predictability. Such studies have mostly implemented using various SDMs, and their predictive power depends on the data availability. Most SDM studies used only abiotic factors like climatic and topographic variables, reflecting microclimatic conditions, to evaluate the effects and vulnerabilities of climate change on a species due to the lack of information on biotic factors and related spatial data. While abiotic variables, such as climate and topography, could be highlighted in regional and global‐scale studies, biotic variables, such as food resources, predators, competitors, etc., have been mostly highlighted at the local‐ and fine‐scale studies (André et al. 2022; Dantas and Fonseca 2023; Huang et al. 2021; Jankowski et al. 2013; Torres‐Romero and Olalla‐Tárraga 2015; Wong et al. 2021). However, it could be species‐specific. Regardless of spatial scales, biotic factors would be crucial information to understand the dynamics of species' distribution that heavily depend on specific food sources (Baer and Gray 2022; Dormann et al. 2018; Wisz et al. 2013). Previous studies, even if limited, showed the importance of biotic factors in predicting species' habitats and distributions (Baer and Gray 2022; Dormann et al. 2018; Wisz et al. 2013). Even though including biotic factors makes the prediction process laborious due to complex interactions among multiple factors, it will increase the model's predictive power and reality, providing better information for management and conservation practices. Our study used biotic variables, the distributions of diet plant species predicted based on the results of greenhouse and field experiments, showing that *P. bremeri* fed on herbaceous plants of the *Sedum* genus, particularly *S. kamtschaticum* and *S. aizoon* (Kim et al. 1999). However, the contributions of *S. kamtschaticum* and *S. aizoon* to the current and future ranges of *P. bremeri* were trivial. The reason could be their extensive distribution in ROK and the possibility that *P. bremeri* is a generalist, which eats locally available herb species, contradicting previous findings. Our study suggests further feeding ecological studies for *P. bremeri* to explore its feeding habits and possible food sources.

We are conscious that we must improve our model predictability to explain real‐world situations. The improvement will be achieved by enhancing model performance and increasing ecological knowledge and data for model parameters. Model performance could also be increased by developing a new model or using hybrid models incorporating existing models to SDM. Understanding the migration network and probabilities of the habitat connectivity could be critical, particularly for small free‐moving insects, such as *P. bremeri*; however, the cellular automata method used in this study is limited to such complex network‐based relationships. Therefore, *P. bremeri*‐specific models, which explain its migration network and habitat connectivity, must be developed and cooperated with SDMs. For this, we need more ecological knowledge and data obtained from field and indoor experimental and theoretical studies. Particularly, diverse ecological studies and surveys on microhabitat analyses, trophic interactions, environmental monitoring, and phenotypic plasticity are required to explore the environmental and trophic niche of *P. bremeri*. However, despite such limitations, the hybrid model, linking land‐use change and dispersal models to SDMs, more realistically predicted the future distributions of *P. bremeri*. Our results also reveal that ensuring strong climate change mitigation policies as well as conservation policies and measures including species‐specific management policies, techniques, and conservation practices are important in conserving endangered species. Our findings generally provide important insights into *P. bremeri* and other endangered species' conservation policies and practices, especially for the endangered species whose future distributions were dominantly influenced by climate change.

## Author Contributions

**Kyung Ah Koo:** conceptualization (lead), data curation (supporting), formal analysis (supporting), funding acquisition (equal), investigation (equal), methodology (equal), project administration (lead), resources (supporting), software (supporting), supervision (lead), validation (supporting), visualization (supporting), writing – original draft (lead), writing – review and editing (lead). **Seon Uk Park:** conceptualization (equal), data curation (lead), formal analysis (equal), investigation (equal), methodology (supporting), resources (lead), software (lead), supervision (supporting), validation (equal), visualization (lead), writing – original draft (equal).

## Conflicts of Interest

The authors declare no conflicts of interest.

## Supporting information


Appendix S1.

## Data Availability

The data supporting the findings of this study are available at https://drive.google.com/drive/folders/1g_4Lk77ysUC36yJ0LcZ0n8N4k7FOVzSq?usp=sharing. Those data include the current and future climate data of the Republic of Korea, the species location data, and R codes for SDM and Miclim modeling. However, the location data for *P. bremeri* is not provided due to the strict prohibition by the domestic law of the Republic of Korea. All other data used and produced in this study, including maps, are presented in Appendix S1. Access to these data will facilitate further research and understanding of the impacts of climate change on endangered butterfly species.
