# Supplementary material for: A Dark Future of Endangered Mountain Species, Parnassius bremeri, Under Climate Change
Source: Ecol Evol. 2025 Apr 1;15(4):e71178. doi: 10.1002/ece3.71178 (PMC11961400; doi:10.1002/ece3.71178)

**Appendix SI**

**Table S1**. Future land-use change predictions under three SSP scenarios. Unit is km^2^.

| **Land covers** | **Current** | **SSP1-2.6** | | **SSP2-4.5** | | **SSP3-7.0** | |
| --- | --- | --- | --- | --- | --- | --- | --- |
|  |  | **2050** | **2100** | **2050s** | **2100** | **2050** | **2100** |
| Urban area | 8,019 | 6,708 | 7,065 | 7,047 | 7,410 | 1,089 | 10,710 |
| Agricultural land | 27,354 | 29,164 | 28,814 | 29,005 | 28,621 | 27,399 | 26,792 |
| Forest | 98,953 | 97,717 | 97,561 | 97,567 | 97,385 | 96,057 | 95,782 |
| Grass | 3,885 | 4,436 | 4,585 | 4,406 | 4,609 | 4,480 | 4,741 |
| Wet land | 1,047 | 308 | 308 | 308 | 308 | 308 | 308 |
| Barren | 2,082 | 2,203 | 2,203 | 2,203 | 2,203 | 2,203 | 2,203 |
| Water | 3,639 | 2,370 | 2,370 | 2,370 | 2,370 | 2,370 | 2,370 |

**Table S2**. Current and future predictions on the areas of suitable habitats for two prey species. Future areas were predicted under three SSP scenarios in association with climate change. Unit is Km^2^.

| **Species name** | **Current** | **SSP1-2.6** | | **SSP2-4.5** | | **SSP3-7.0** | |
| --- | --- | --- | --- | --- | --- | --- | --- |
|  |  | **2050** | **2100** | **2050** | **2100s** | **2050s** | **2100s** |
| *S. kamtschaticum* | 24,284 | 28,455 | 30,437 | 29,706 | 29,911 | 36,629 | 41,800 |
| *S. aizoon* | 26,955 | 71,635 | 80,398 | 79,389 | 82,456 | 84,912 | 89,344 |

**Appendix SI**

**Figure S1**. Current land use cover map with seven categories (NEINS; <https://www.neins.go.kr/Index>).


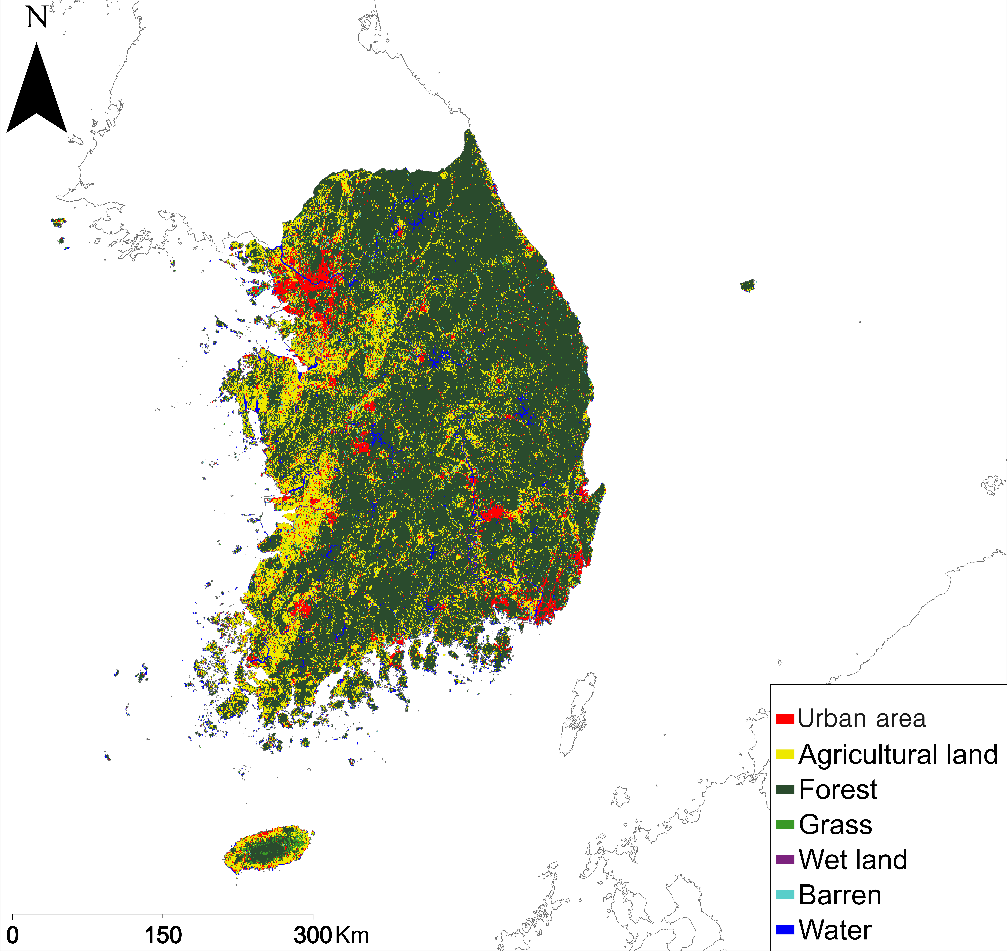


**Figure S2**. Future predictions of land-use changes in 2050 and 2100 under three SSP scenarios. (a) presents the land-use change prediction in 2050 under SSP1, (b) 2100 under SSP1, (c) 2050 under SSP 2, (d) 2100 under SSP 2, (e) 2050 under SSP3, and (f) 2100 under SSP3 (Song et al., 2018).


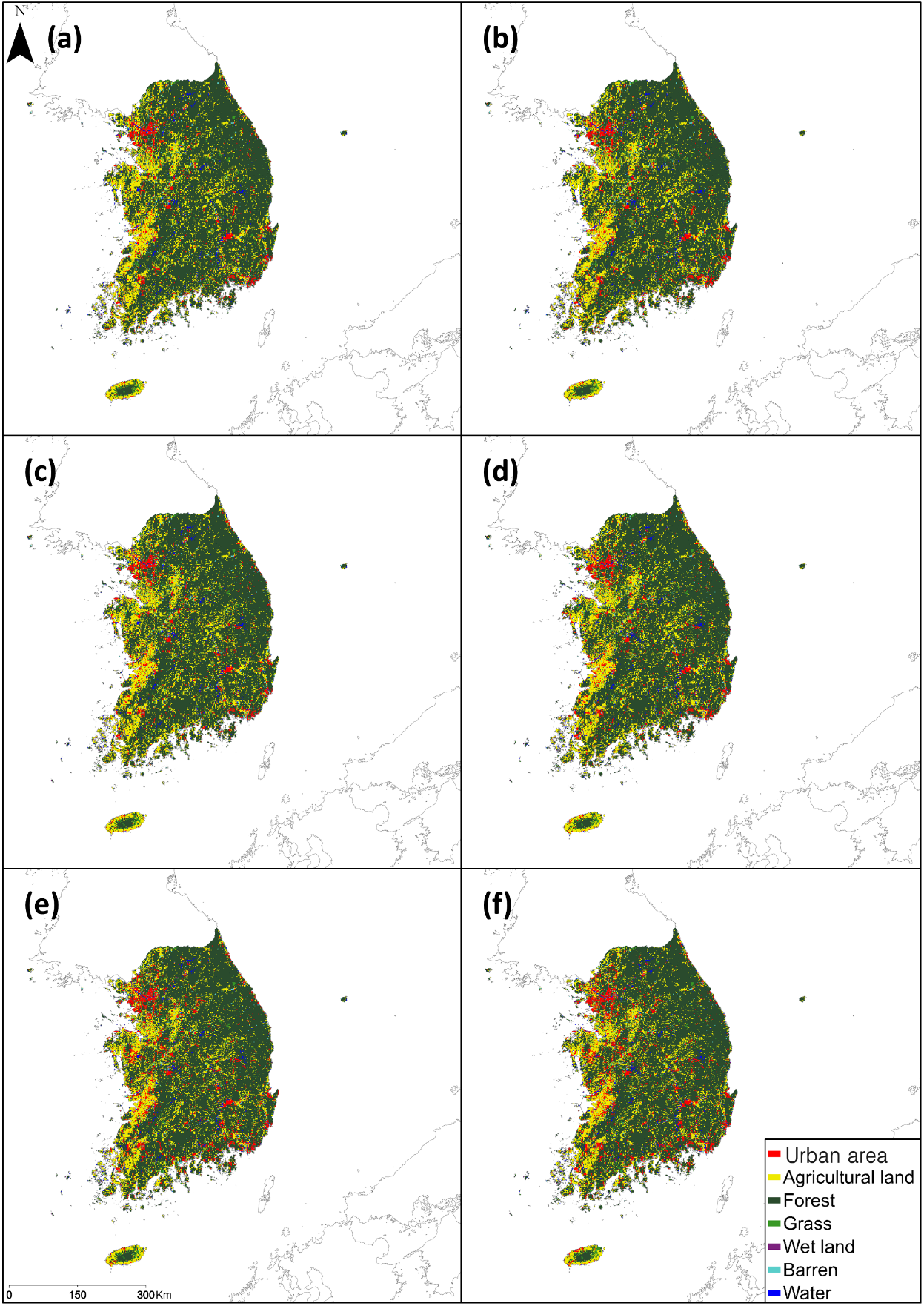


**Figure S3**. The current projected range (the suitability > 0.555) and habitat suitability of *S. kamtschaticum* are presented by the probability of species presence*.* The colored areas present suitable for presence, indicating the current range, and the white colored area unsuitable habitats, indicating species absence.


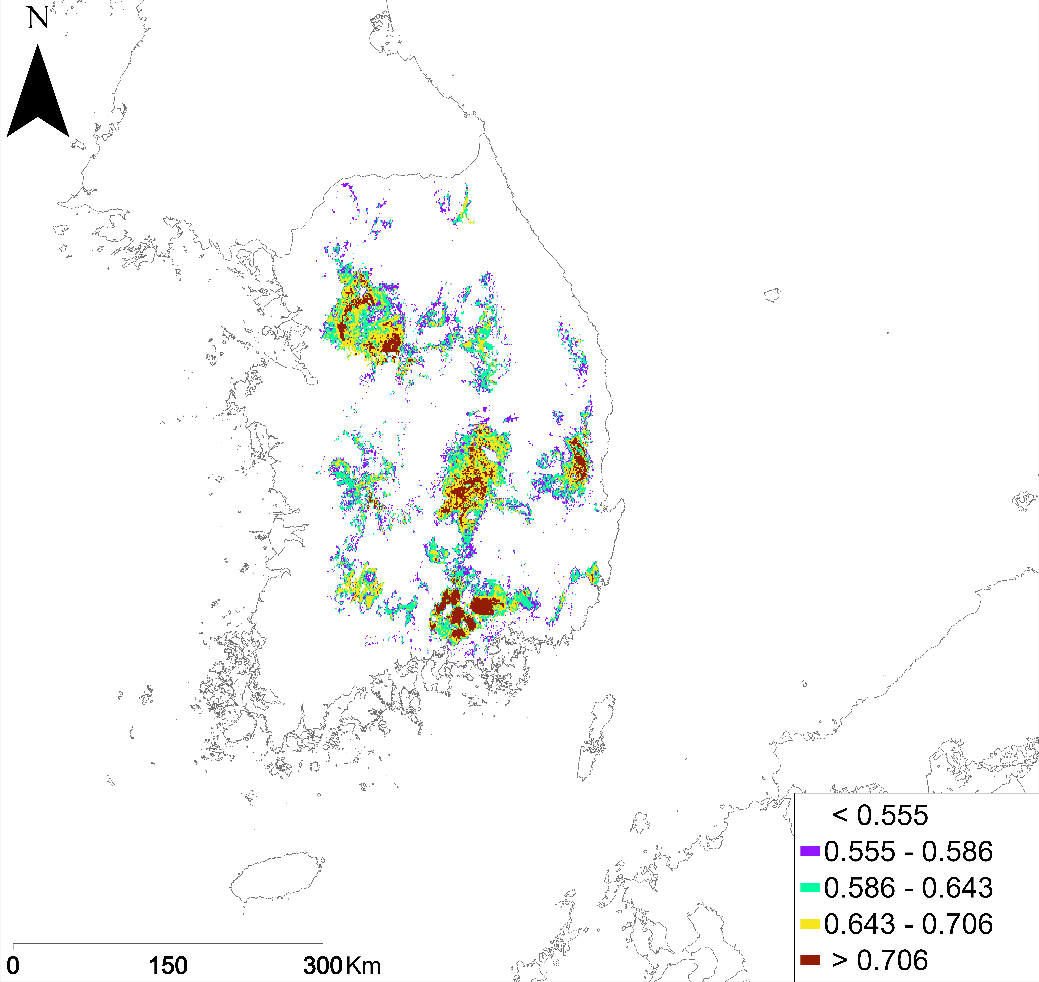


**Figure S4**. Future predictions of habitat suitability and distributional range for *S. kamtschaticum* in 2050 and 2100 under three scenarios. Figure (a) shows the future range (the colored areas, the suitability > 0.555) and the distribution of habitat suitability in 2050 under SSP1-2.6, (b) 2100 under SSP1-2.6, (c) 2050 under SSP2-4.5, (d) 2100 under SSP2-4.5, (e) 2050 under SSP3-7.0, (f) 2100 under SSP3-7.0.


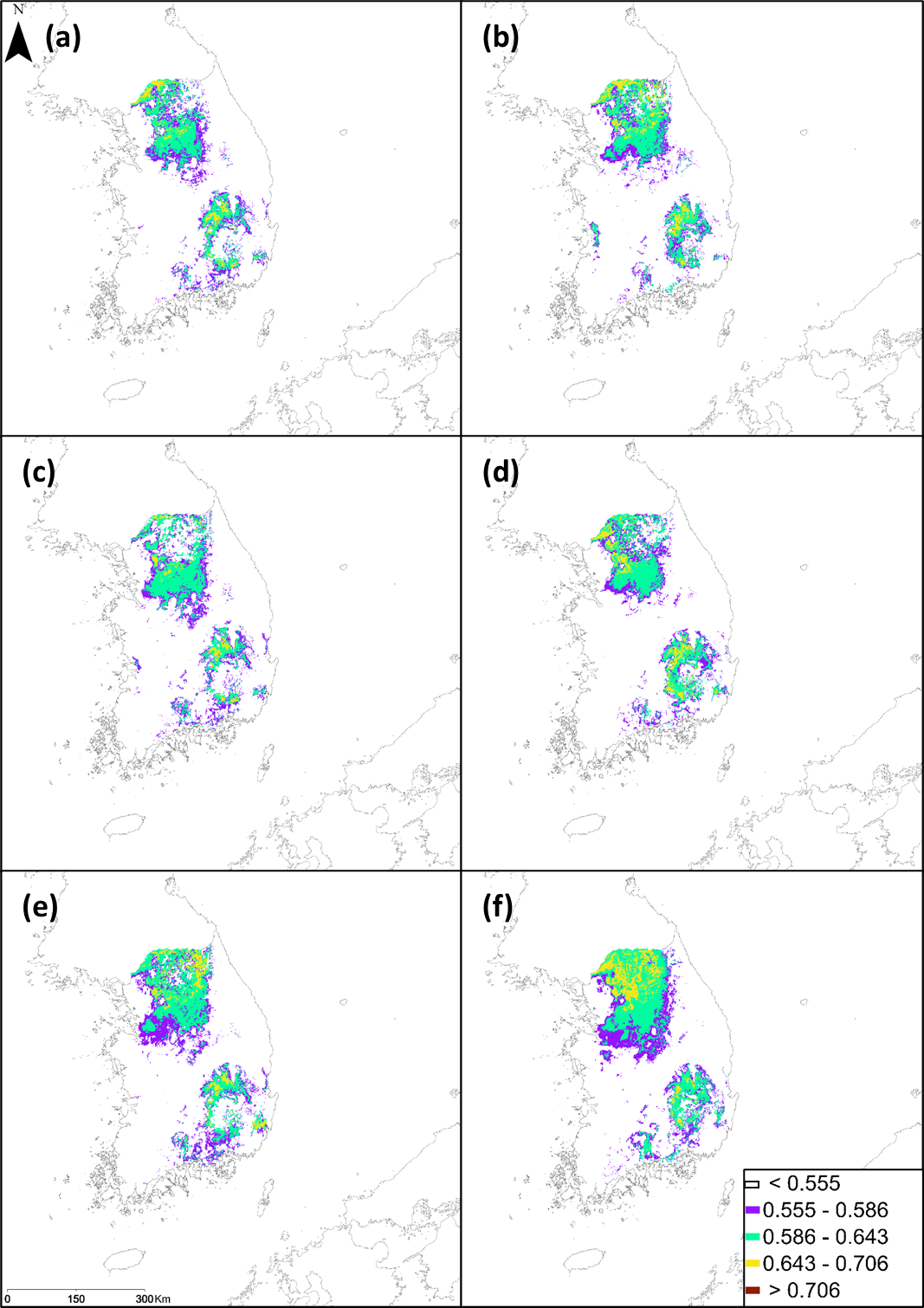


**Figure S5.** The current projected range (the suitability > 0.360) and habitat suitability of *S. aizoon* presented by the probability of species presence*.* The colored areas present suitable for presence, indicating the current range, and the white colored area unsuitable habitats, indicating species absence.


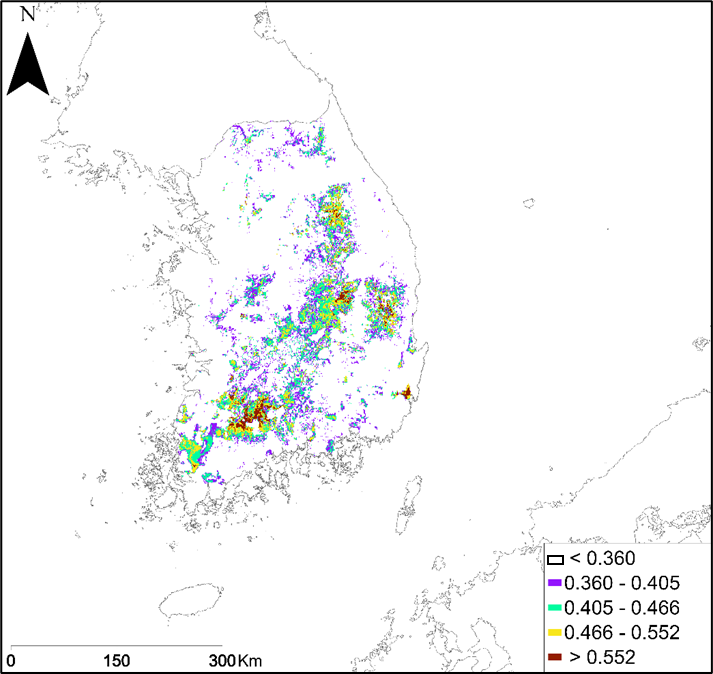


**Figure S6.** Future predictions of habitat suitability and distributional range for *S. aizoon* in 2050 and 2100 under three scenarios. Figure (a) shows the future range (the colored areas, the suitability > 0.360) and the distribution of habitat suitability in 2050 under SSP1-2.6, (b) 2100 under SSP1-2.6, (c) 2050 under SSP2-4.5, (d) 2100 under SSP2-4.5, (e) 2050 under SSP3-7.0, (f) 2100 under SSP3-7.0.


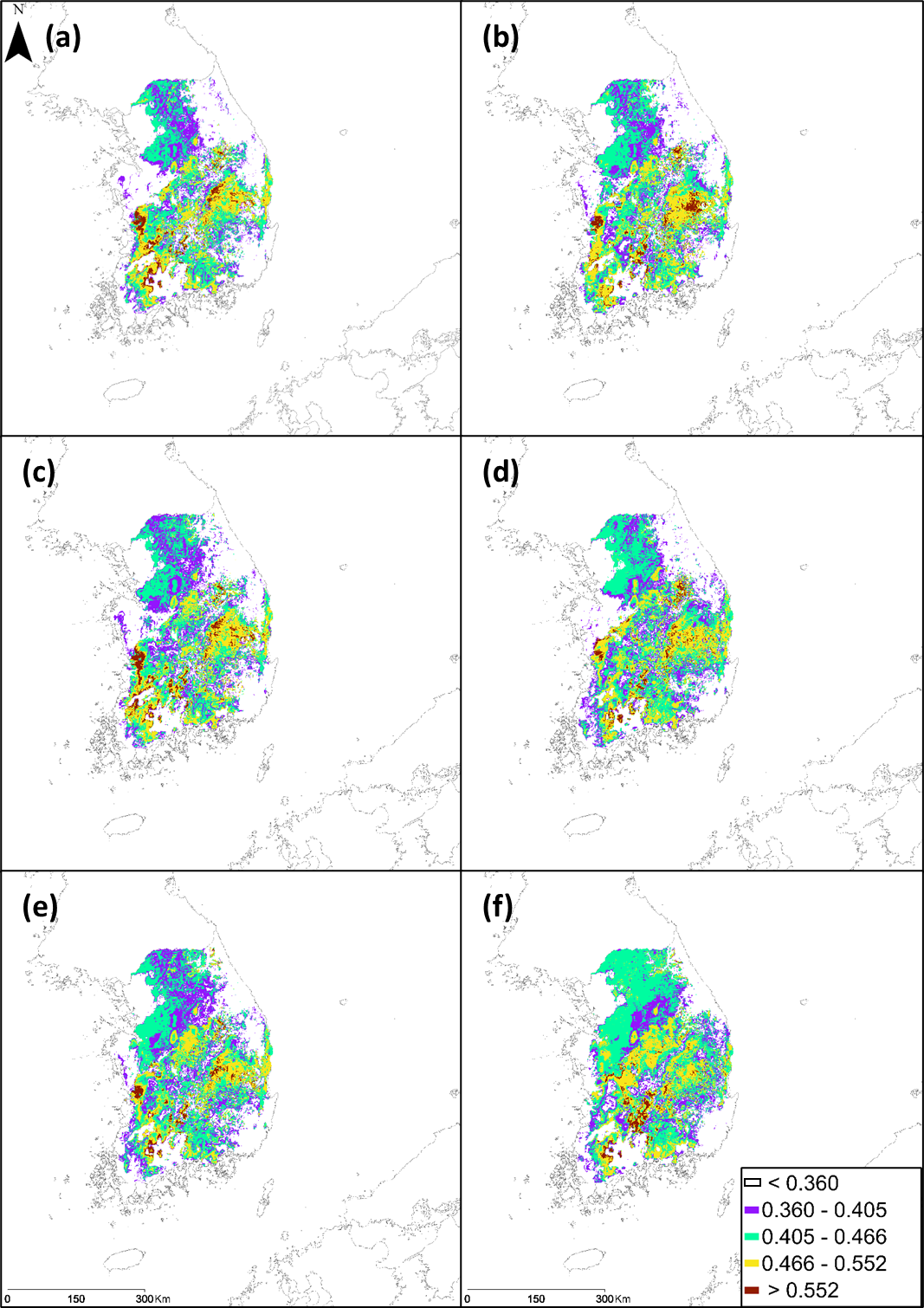

Supplement: Supplementary file 1 — Appendix S1. [file ECE3-15-e71178-s001.docx]
